# PARTNERSHIPS THAT WORK: AN ACADEMIC AND STATE COLLABORATION IN KANSAS

**DOI:** 10.1093/geroni/igz038.2054

**Published:** 2019-11-08

**Authors:** Gayle Doll, Migette Kaup, Laci Cornelison

**Affiliations:** Kansas State University, Manhattan, Kansas, United States

## Abstract

Academic partnership with state government may be a researcher’s dream or a tremendous burden. This presentation demonstrates the perspectives of university personnel as well as government leaders when contracts and grants are issued for the provision of research and services. Opportunities and barriers for researchers will be discussed.

